# Detection of autoantibodies in severe COVID-19 patients two years after hospital discharge

**DOI:** 10.1590/1414-431X2025e14927

**Published:** 2025-11-14

**Authors:** E.M.B. Hi, C.C.R. Bianchi, R.B. Gritte, P.H.A. Klauss, N.F.S.M. Leal, I.S. de Oliveira, M.F.C.B. de Barros, F.G. Soriano, R. Curi, M.C.C. Machado

**Affiliations:** 1 Laboratório de Emergências Clínicas, Departamento de Clínica Médica, Faculdade de Medicina, Universidade de São Paulo, São Paulo, SP, Brasil; 2 Faculdade de Ciências Médicas de Santos, Centro Universitário Lusíada, Santos, SP, Brasil; 3 Curso de Biomedicina, Centro Universitário Lusíada, Santos, SP, Brasil; 4 Faculdade de Ciências da Saúde, Universidade do Oeste Paulista, Guarujá, SP, Brasil; 5 Faculdade de Farmácia, Universidade Federal da Bahia, Salvador, BA, Brasil; 6 Programa de Pós-Graduação Interdisciplinar em Ciências da Saúde, Universidade Cruzeiro do Sul, São Paulo, SP, Brasil; 7 Centro de Ensino, Instituto Butantan, São Paulo, SP, Brasil; 8 Pró-reitoria de Pesquisa e Pós-graduação, Universidade de Marília, Marília, SP, Brasil

**Keywords:** Severe COVID, Autoantibodies, Antinuclear antibody, Rheumatoid factor, Anti-cyclic citrullinated peptide

## Abstract

After SARS-CoV-2 infection, severe COVID-19 may develop with persistent sequelae, even after hospital discharge. This condition may result from tissue damage or immune alterations caused by the virus, including immune dysregulation, hyperinflammation, loss of immune tolerance, excessive neutrophil extracellular trap (NET) production, and antibody cross-reactivity (molecular mimicry), which can promote autoantibody development. This study evaluated autoantibody expression in patients with long COVID-19 and its potential relationship with symptoms. Conducted in Baixada Santista, São Paulo, Brazil, the study involved 55 participants aged 21-85 years who had tested positive for SARS-CoV-2. Blood samples were collected two years post-discharge, and serum was analyzed for inflammatory and autoimmune markers, including antinuclear antibody (ANA), rheumatoid factor (RF), anti-cyclic citrullinated peptide (anti-CCP), procalcitonin (PCT), Venereal Disease Research Laboratory test (VDRL), and C-reactive protein (CRP). Results were compared to a control group of 21 individuals who never tested positive for COVID-19. Among severe COVID-19 patients, 26 reacted to ANA, 16 to VDRL, 2 had elevated RF, 12 had increased PCT, and 11 had high CRP, whereas the control group showed no reactive results. Anti-CCP values were not significant. Findings suggest that hyperinflammation may contribute to autoimmunity, particularly in cases of reactive ANA levels, linking COVID-19 symptoms to autoimmune responses.

## Introduction

COVID-19, the clinical manifestation of the infection by the SARS-CoV-2 virus, was first reported in Wuhan, China, in December 2019. It was declared a pandemic by the World Health Organization (WHO) on March 11, 2020. Although it initially manifests as an acute respiratory syndrome, COVID-19 can progress rapidly and have systemic impact. The degree of involvement ranges from asymptomatic to severe illness and death (1).

On average, 10% of infected individuals develop the so-called long COVID-19 syndrome, which includes the complications of the disease; in hospitalized patients the incidence of 50-70%. The most common pathologies include cardiovascular, respiratory, thrombotic, and cerebrovascular alterations, resulting in symptoms such as palpitations, dyspnea, coughing, fatigue, memory loss, insomnia, and even mental confusion (2).

The primary hypotheses for the origin of complications in patients hospitalized due to COVID-19 infection are long-term tissue damage affecting the lungs, brain, and heart, as well as pathological inflammation resulting from viral persistence, immune dysregulation, or autoimmunity. Viral particles have been found to persist for 4 months after the initial infection through positive qPCR tests. These antigens activate the immune system for extended periods, leading to autoimmune states (1).

Autoimmune phenomena occur when antibodies recognize the body's tissues or cells as antigens. Although the etiology has yet to be fully elucidated, factors such as genetic predisposition, bacterial, viral, or parasitic infections, hormonal issues, and immune system dysregulation are the primary triggers (3). In long COVID, autoimmune disorders are triggered through three main mechanisms: molecular mimicry between viral proteins and self-antigens, temporary impairment of the immune response, and subsequent inadequate immune reconstitution in individuals with a predisposition to autoimmunity (4).

The biochemical similarity between SARS-CoV-2 structures and host self-structures, such as resemblance between the spike protein and mitochondrial peptides, favors immunological cross-reactions, potentially leading to autoimmune sequelae as a pathological consequence (4).

The impairment of the innate and acquired immune response is a risk factor because it causes a loss of tolerance to self-antigens. The invasion of cells by the virus naturally triggers the defense system, redistributing lymphocytes to the affected area and stimulating inflammatory reactions, which result in transient lymphopenia. This change has a significant impact, as one of the functions of lymphocytes is their sentinel role in preventing antibodies from targeting self-antigens. Consequently, with the loss of tolerance to self-antigens, the production of autoantibodies is stimulated (5).

Inadequate immune reconstitution can occur when lymphocyte levels are re-established during recovery. In addition, issues such as genetic predisposition often lead to a new uncontrolled immune response characterized by excessive cytokine production and subsequent autoimmune alterations (5), which may lead to a long-term inflammatory response (6).

The incidence of autoimmune diseases was measured in patients who had COVID-19 (4-6), showing that the risk of developing diseases such as Behçet's and mixed connective tissue disease is considerably higher for individuals who have already contracted the disease compared to the control group. In these patients, autoantibodies such as rheumatoid factor (RF), anti-nuclear antibody (ANA), anti-double-stranded DNA (anti-DNA), and anti-cardiolipin were detected, supporting the presence of autoimmunity in this population (7). Tesch et al. (8) investigated 641,000 patients with COVID-19 and reported that 42.6% were more likely to develop autoimmunity compared to healthy individuals.

In the present study, we investigated the presence of autoantibodies as well as inflammatory markers (C-reactive protein - CRP), in patients hospitalized due to severe COVID-19. All analyses were conducted on blood samples collected two years after hospital discharge, allowing for the evaluation of long-term immunological alterations.

## Material and Methods

### Study participants and data collection

The study was carried out along the São Paulo shore - Baixada Santista, SP - and included patients treated for COVID-19 complications at Santa Casa de Santos and Hospital Municipal Guilherme Álvaro.

The inclusion criteria were a positive SARS-CoV-2 qPCR test result from a nasopharyngeal swab (n=55) and hospital admission between 2020 and 2021. Exclusion criteria were individuals with HIV, chronic hepatitis, cancer, and/or autoimmune or inflammatory diseases, as well as those undergoing continuous treatment with anti-allergic medications (antihistamines or corticosteroids) and/or anti-inflammatory drugs (nonsteroidal or corticosteroids). All exclusion criteria were confirmed at the time of blood sample collection.

A health and well-being questionnaire was administered to individuals in the COVID-19 group. The questionnaire was adapted from the ISARIC Global COVID-19 Follow-Up Level 1 Tier 1 Case Report Form, version 1.2. Based on the responses, the most frequently reported symptoms that emerged following hospital discharge due to COVID-19 were identified. A total of 44 patients completed the questionnaire.

The control group included individuals with similar age and gender distribution to the study group who had no history of SARS-CoV-2 infection. This was confirmed by negative results in all diagnostic tests performed up to the time of blood sample collection, including nasopharyngeal swab sample qPCR and rapid tests for anti-SARS-CoV-2 antibodies and SARS-CoV-2 antigen (n=21).

### Blood collection

The samples were collected 2 years after hospital discharge. Approximately 10 mL of blood from the antecubital vein was collected into BD vacutainer dry tubes (Becton Dickinson, USA). After collection, the serum was obtained by centrifuging the samples (2000 *g* for 15 min at room temperature).

### Biomarker determination

The rheumatoid factor (RF) and C-reactive protein (CRP) kits were acquired from Labtest Diagnóstica S.A. (Brazil) and run on an automated DIRUI CS-240 analyzer (China), following the manufacturer's instructions.

An anti-cyclic citrullinated peptide (anti-CCP) ELISA kit (Euroimmun, Germany) was used to detect the anti-CCP marker, according to the kit's instructions.

The human procalcitonin ELISA Kit (Thermo Fisher Scientific Inc., USA) was used to assess procalcitonin (PCT) levels.

According to the manufacturer's information, the ANA-HEP2 IFT kit (Euroimmun), diluted 1:80, was used to detect antinuclear antibodies (ANA). The method has approximately 180 possible self-antigens for detection, expressed through different fluorescence patterns. This assay is considered the gold standard for ANA screening.

The Venereal Disease Research Laboratory (VDRL) test was performed using the VDRL/Syphilis kit (Wama Diagnóstica, Brazil), with a final dilution of 1:8, according to the manufacturer's instructions.

### Statistics

Statistical analyses were performed using GraphPad Prism version 10 (GraphPad Software, USA). Data distribution was assessed using the Shapiro-Wilk test, which indicated that the variables did not follow a normal distribution. Therefore, COVID-19 and control groups were compared using the non-parametric Mann-Whitney U test. Results were considered statistically significant when P<0.05.

## Results

Information such as gender, age, and comorbidities (e.g., hypertension, diabetes, obesity, asthma, and dyslipidemia) of the participants was collected from the medical records made available by the hospitals (Table 1).

**Table 1:** Characterization of patients.

	Control (n=21)	COVID-19 (n=55)
Gender, n (%)		
Female	16 (76.19%)	25 (45.45%)
Male	5 (23.81%)	30 (54.55%)
Age		
Mean (SD)	44.82 (15.42)	45.96 (13.57)
Median (min-max)	46 (22-74)	47 (21-85)
Comorbidities, n (%)		
Obesity	-	9 (16.36%)
Asthma	-	2 (3.64%)
Dyslipidemia	-	1 (1.82%)

The results of plasma autoimmune markers, such as ANA, VDRL, RF, and anti-CCP, and inflammatory markers PCT and CRP are shown in Table 2.

**Table 2:** General results of the control and COVID-19 groups.

Markers	Control	COVID-19
ANA		
Positive	-	26 (47.27%)
Negative	21 (100%)	29 (52.73%)
VDRL		
Positive	-	16 (29.09%)
Negative	21 (100%)	39 (70.91%)
CRP		
≥6.0 mg/L	-	11 (20%)
<6.0 mg/L	21 (100%)	44 (80%)
Mean (SD)	0.99±1.82	4.24±6.99
PCT		
≥500 pg/mL	-	12 (21.82%)
<500 pg/mL	21 (100%)	43 (78.18%)
Mean (SD)	61±26	693±1540
RF		
>20 U/mL	-	2 (3.64%)
<20 U/mL	21 (100%)	53 (96.36%)
Mean (SD)	5.45±11.77	6.27±7.47
Anti-CCP		
≥5.0 U/mL	-	-
<5.0 U/mL	21 (100%)	55 (100%)
Mean (SD)	1.345±0.46	1.187±0.28

ANA: antinuclear antibodies; VDRL: Venereal Disease Research Laboratory test; CRP: C-reactive protein; PCT: procalcitonin; RF: rheumatoid factor; Anti-CCP: autoantibodies against citrullinated peptides.

**Figure 1:**
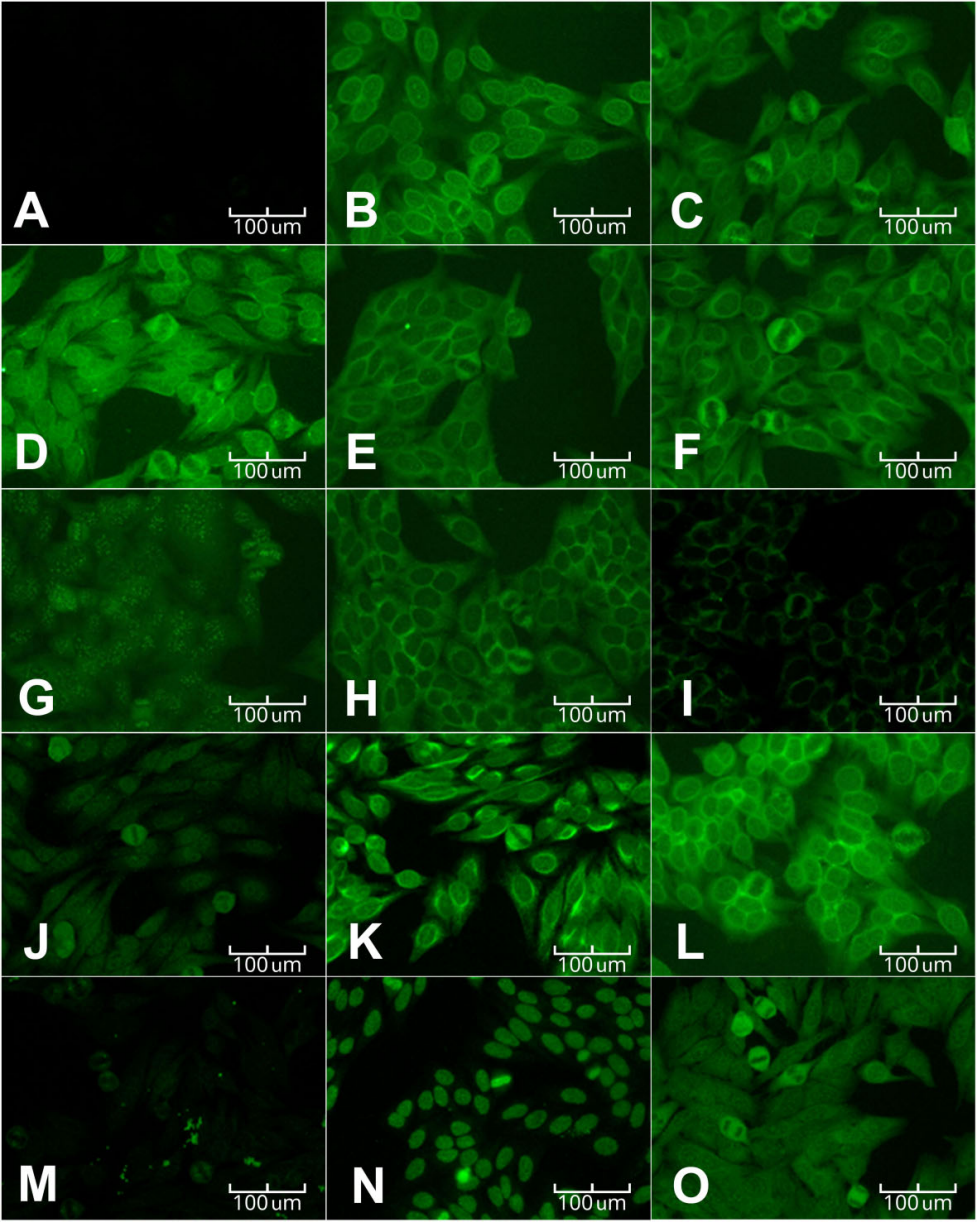
Antinuclear antibody (ANA) patterns found in patients. Images obtained using fluorescence microscopy (scale bar, 100 μm). **A**, Negative reaction; **B**, Homogeneous Nucleolar; **C**, Nucleolar/Roads and Rings; **D**, Roads and Rings; **E**, Cytoplasmatic Dense Fine-speckled/Nucleolar; **F**, Cytoplasmatic Dense Fine-speckled; **G**, Centromere; **H**, Fine-speckled with Discrete Dots; **I**, Reticular/Antimitochondrial Antibody (AMA); **J**, Nuclear Fine-speckled; **K**, Fibrillar Filamentous; **L**, Punctate Nuclear Envelope; **M**, Mitotic Spindle Pattern (NuMA-like); **N**, Nuclear Dense Fine-speckled; **O**, Cytoplasmatic Dense Fine-speckled and Multiple Nuclear Dots.

Twenty-six severe COVID patients had a positive result for ANA reactivity at the 1:80 dilution. In contrast, all the 21 patients in the control group did not present fluorescence. As shown in Figure 1, 14 different ANA fluorescence patterns were observed. The most frequently identified pattern was the nuclear fine-speckled pattern (Table 3).

**Table 3:** Frequency of antinuclear antibody (ANA) patterns.

ANA Patterns	Clinical correlation	Frequency
Nuclear Fine-speckled	Sjögren's syndrome (SSj), systemic lupus erythematosus (SLE), systemic sclerosis (SSc) and SSc-AIM overlap syndrome (autoimmune myositis)	7
Rods and Rings	SLE	5
Nuclear membrane Speckled	SARD (Systemic autoimmune rheumatic diseases) and primary biliary cholangitis (PBC)	2
Homogeneous nucleolar	SSc, SSc-AIM overlap syndrome	2
Cytoplasmic fine-speckled dense	SLE, anti-synthetase syndrome and polymyositis	1
Centromeric	systemic sclerosis (SSc)	1
Cytoplasmic Speckled with isolated dots	SARD, SjS (Stevens-Johnson syndrome), associated with neurological symptoms, and systemic sclerosis	1
Cytoplasmic Speckled reticulate	Chronic obstructive pulmonary disease, pulmonary fibrosis, SARD	1
Cytoplasmic fibrillar filamentary	SARD, SjS, SLE, undifferentiated connective tissue disease (UCTD), limited SSc or rheumatoid arthritis (RA)	1
Mitotic spindle (NuMA-2)	Nonspecific for autoimmunity (common in healthy individuals)	1
Nuclear fine-speckled dense	SSc, SSc-AIM overlap syndrome, SLE	1
Nucleolar rods and rings	SLE, anti-synthetase syndrome, SSc, SSc-AIM overlap syndrome	1
Cytoplasmic fine-speckled dense and homogeneous nucleolar	SLE, anti-synthetase syndrome, AIM, and other inflammatory conditions.	1
Cytoplasmic fine-speckled dense and multiple nuclear dots	Sjögren's syndrome, SLE, systemic sclerosis, and SSc-AIM overlap syndrome (autoimmune myositis)	1

As shown in Table 2, there were 16 VDRL-reactive samples from the COVID group, 7 with titration 1:2, 6 with titration 1:4, and 3 with titration 1:8. The 21 control samples showed non-reactive results. All samples that displayed VDLR reactivity were submitted to the confirmatory test for syphilis (FTA-Abs) and the rapid treponemal test, which showed unreactive results for treponemal antibodies.

**Figure 2:**
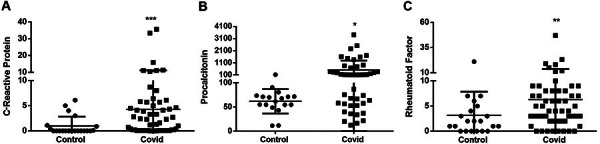
Ratio of biomarkers in the control group and in the COVID-19 group. **A**, C-reactive protein; **B**, procalcitonin; **C**, rheumatoid factor. Horizontal bars represent the median (central thick bar) and interquartile range (upper and lower thinner bars). *P<0.05; **P<0.01; ***P<0.001. Mann-Whitney U test.

CRP levels were above 6 mg/dL in 11 samples from the COVID group. The results suggest a correlation between ANA and elevated CRP levels, as individuals with increased CRP levels exhibited positive fluorescence patterns. As shown in Figure 2A, the average CRP level of the COVID group was 4 times higher than that of the control group (P<0.05).

PCT levels were above 500 pg/mL in 12 samples from the COVID group, which correlates with systemic inflammation. The mean PCT level of the COVID group was 10 times higher than that of the control group (P<0.01), as shown in Figure 2B.

The RF level is considered reactive when it is above 20 IU/mL, as observed in two patients in the COVID group, with only one also expressing positivity in other tests (reactive ANA and elevated CRP). As shown in Figure 2C, both groups presented similar results, with the COVID-19 group having an average of 6.27 IU/mL and the control group, 5.45 IU/mL (P<0.001).

The anti-CCP level measured by ELISA is considered positive when above 5 U/mL. Based on this criterion, none of the samples from the COVID or control groups were positive. The groups tested had an average below 2 U/mL (Table 2).

A total of 44 patients completed the health and well-being questionnaire. The most frequently reported symptoms persisting after two years of the acute phase of COVID-19 included headache (22/44; 50%), muscle weakness in the arms or legs (15/44; 34.09%), confusion or lack of concentration (12/44; 27.27%), tingling or “pins and needles” sensation (12/44; 27.27%), shortness of breath or breathlessness (11/44; 25%), and persistent muscle pain (11/44; 25%). Additional symptoms reported with lower frequency included joint pain or swelling (10/44; 22.73%), palpitations (8/44; 18.18%), pain on breathing (4/44; 9.09%), and skin rashes (5/44; 11.36%). Among the patients who reported symptoms, 13 tested positive for antinuclear antibodies (ANA), suggesting a possible association between COVID symptoms and autoimmune responses.

Among the individuals in the COVID-19 group, only five had received a COVID-19 vaccine. Four of them were vaccinated before hospitalization, and one individual was vaccinated after discharge from the hospital. Of the four patients vaccinated before COVID-19 infection, only two exhibited a positive antinuclear antibody (ANA) result. One of these individuals had received the CoronaVac (Sinovac Biotech, China) vaccine, while the other had received the AstraZeneca (Brasil) vaccine.

## Discussion

The present study evaluated autoimmune and inflammatory markers in individuals two years after hospitalization due to severe COVID-19. Among the 55 patients in the COVID-19 group, only five had received a COVID-19 vaccine, as indicated above. Due to the small number of vaccinated individuals and the minimal overlap with autoantibody positivity, it is unlikely that vaccination had a significant influence on the findings. Thus, it is plausible that the observed autoimmune markers are associated with the infection itself rather than an immunization response.

The control group consisted of 21 individuals who tested negative for SARS-CoV-2 by qPCR, antigen, and antibody tests during the pandemic period. Although efforts were made to match the age and sex distribution with the COVID-19 group, a balanced gender distribution could not be achieved due to recruitment limitations, particularly the difficulty in identifying individuals who met all inclusion and exclusion criteria without prior infection. Additionally, the possibility of prior asymptomatic SARS-CoV-2 infection in the control group cannot be entirely excluded, representing a potential limitation of the study. Despite rigorous screening, undetected past infections could not be definitively ruled out due to the limitations of testing sensitivity and the lack of more specific immunological assays. However, the complete absence of autoantibody reactivity in the control group strengthens the hypothesis that the immune alterations identified in the COVID-19 group were related to prior SARS-CoV-2 infection, rather than baseline background prevalence.

### Autoimmunity and inflammation biomarkers

Of the 55 samples collected from the COVID group, 47.3% had reactive ANA, 29.1% had reactive VDRL, 20% had elevated CRP values, 21.8% had altered PCT levels, and 3.6% showed reactive RF. In contrast, no samples from the control group had reactive results.

#### ANA

The ANA (FAN-HEp-2) method enables the testing of autoantibodies against a series of cellular antigens expressed through distinct fluorescence patterns, classified as anti-cellular (AC) patterns by the International Consensus on Antinuclear Antibodies (ICAP), which suggests a particular antibody. However, it is a triage test, only indicating the need for more specific tests when reactive (9).

The Brazilian consensus for autoantibody research in HEp-2 cells considers this titration ideal for testing purposes. Moreover, it has been used internationally, as seen in the study by Pascolini et al. (10), in which 33.3% of the results exhibited reactivity at a 1:80 dilution.

The nuclear fine-speckled pattern (AC-4), more frequently found in the sample group (27%), was also the most prevalent in the study by İnal et al. (11). This pattern is clinically associated with autoimmune diseases such as systemic lupus erythematosus (SLE), systemic sclerosis (SSc), and SSc with autoimmune myositis overlap syndrome (SSc-AIM). Among the seven reactive samples, three patients exhibited symptoms consistent with SLE, including joint pain or swelling and a tingling (“pins and needles”) sensation, and one patient showed muscle weakness indicative of AIM.

Rods and rings (AC-23) pattern was the second most frequently observed pattern (19.23%). This finding is consistent with İnal et al. (11), who reported that the positivity rate for this pattern was 2.16 times higher in the post-COVID period than in the pre-COVID period, particularly in younger patients. This result suggests that the development of this autoantibody may be associated with SARS-CoV-2 infection. Such fluorescence indicates SLE, characterized by joint pain and skin rashes, symptoms in three of the five patients with this pattern.

The punctate nuclear envelope (AC-12) pattern, the homogeneous nucleolar (AC-8) pattern, the centromeric (AC-3) pattern, and the reticular/ antimitochondrial antibody (AMA) (AC-21) pattern are related to SSc. Studies have already presented cases of patients who developed SSc after SARS-CoV-2 infection, and the case reported by Chandra et al. (12) expressed anti-centromeric antibodies.

The fibrillar filamentous (AC-16) pattern can be found in cases of SARDs but is often associated with non-immune conditions, such as pulmonary fibrosis and chronic obstructive pulmonary disease. The nuclear fine-speckled dense pattern (AC-2) is also nonspecific for autoimmunity, with a prevalence in healthy individuals ranging from 0.78 to 16.4%, depending on the population studied (13).

The mitotic spindle (NuMA-like; AC-26) pattern is primarily observed in cases of primary Sjögren's syndrome and systemic lupus erythematosus (SLE), being the only ANA pattern detected in 60% and 50% of cases, respectively (14).

The cytoplasmic dense, fine-speckled pattern (AC-19) has a clinical relationship with SARDs, especially in patients with anti-synthetase syndrome (ASS), a subset of AIMS. According to the study by Phillips et al. (15), there has been a significant increase in the incidence of ASS following COVID-19. For example, three diagnoses were made between March and July 2020 in the same hospital in the United Kingdom, where only six had been made in the previous 15 years, resulting in a 15-fold increase in the annual diagnosis rate.

The cytoplasmic fine-speckled pattern with discrete dots in the same sample is a multiple pattern: when two or more antibodies with different targets are found in the same patient, it is possible to identify them (16).

Multiple nucleolar rods and rings, as well as cytoplasmic dense fine-speckled nucleolar patterns, were present in one patient each, whose nonspecific muscle symptoms (weakness, numbness, and difficulty walking or climbing steps) were compatible with SSc and AIM. The antibodies characteristic of myositis include PM-Scl, expressed in a nucleolar pattern, and anti-synthetase, described in a cytoplasmic dense fine-speckled pattern; the combination of these antibodies reinforces this scenario (17).

Cytoplasmic dense fine-speckled/multiple nuclear dots are the final example of multiple patterns observed in a patient with symptoms corresponding to SLE, such as nodules or rashes all over the body and muscle symptoms suggestive of AIM. The different antibodies in this sample support the divergent diagnostic hypotheses (16).

Among the 26 patients with reactive results, 13 had symptoms compatible with the clinical suspicions indicated by the ANA patterns (Table 4).

**Table 4:** Symptoms reported two years after COVID-19 infection.

Symptom	n (%)
Headache	22 (50.00%)
Shortness of breath/ breathlessness	11 (25.00%)
Pain on breathing	4 (9.09%)
Palpitations (accelerated heartbeat)	8 (18.18%)
Weakness in arms or legs/muscle weakness	15 (34.09%)
Persistent muscle pain	11 (25.00%)
Joint pain or swelling	10 (22.73%)
Tingling or “pins and needles” feeling	12 (27.27%)
Confusion/lack of concentration	12 (27.27%)
Skin rashes	5 (11.36%)

Data extracted from the health and well-being questionnaire (n=44).

It is reported that the presence of ANA is correlated with symptoms of long COVID, including neurological symptoms, body aches, and dyspnea (18).

#### VDRL

The VDRL test is a non-treponemal, low-cost, and complex exam used to screen for syphilis. It uses cardiolipin and lecithin antigens adsorbed on cholesterol crystals to detect anti-cardiolipin and anti-lecithin autoantibodies in the patient's serum. However, as it is a highly sensitive method, it can react to other antibodies and give false-positive results in autoimmune diseases as a direct consequence of SARS-CoV-2 infection or in infections such as influenza and tuberculosis, mainly during the acute phase.

Of the 26 ANA-reactive samples, 16 patients, all from the COVID group, also reacted to VDRL (61.5%). After these analyses, the samples were submitted to the FTA-Abs treponemal test (confirmatory for syphilis), which yielded non-reactive results for treponemal antibodies in all cases. Considering that the samples were collected 2 years after infection, the hypothesis that best justifies these results is the presence of autoantibodies. In the study by Al Attia (19), 24.5% of patients with SLE produced false-positive results using this methodology.

Antiphospholipid antibodies (aPL), such as anti-β2-glycoprotein I and anti-cardiolipin, can be transiently present in various infections, including hepatitis C and HIV, and have recently been identified in COVID-19 cases. A study conducted by Butt et al. (20) (1,159 patients infected with SARS-CoV-2) found that the prevalence rate of aPL was 46.8%. However, the autoantibodies were not detected after 3 months. Altmann et al. (21) reported the presence of antiphospholipid antibodies in the serum of patients hospitalized due to COVID-19, which is related to antiphospholipid syndrome (an autoimmune disorder) and coagulation disorders.

The presence of aPL antibodies in patients with long COVID, even at low titers (1:2, 1:4, 1:8), reinforces the hypothesis that autoimmunity may develop after infection. The study by Ahmed et al. (22) reports a case compatible with this hypothesis, where a blood donor for 5 years presented a reactive VDRL result 3 months after being diagnosed with COVID-19, reporting no sexual contact in the last year and no high-risk sexual behavior. The test remained reactive for another 16 weeks, totaling 7 months since the SARS-CoV-2 infection until the test was negative. This result supports the possibility of routinely carrying out the VDRL test for screening long COVID patients for suspected autoimmune diseases.

#### CRP

CRP is an inflammatory biomarker widely used in the COVID-19 pandemic as a marker of disease severity. It was also the most sensitive marker of a worsening condition of the patient during the acute phase and even of the persistence of symptoms after the infection had resolved. The presence of high CRP levels, together with other markers, months after hospital discharge confirmed the persistence of the long-term inflammatory process in long COVID (23).

Despite being a nonspecific marker, the intensity of the inflammatory response was evaluated by Levinson et al. (24) in various viral infections that affect the respiratory tract, including influenza A, influenza B, and respiratory syncytial virus. The authors showed that patients with SARS-CoV-2 are more likely to have significantly higher CRP values upon hospital admission. This observation is evidence of the exacerbated inflammation that COVID-19 causes compared to other viral respiratory diseases.

In our study, 11 patients in the COVID group had results above the reference value (6 mg/dL). Of these, 63.4% tested positive for ANA. Son et al. (25) also showed a positive correlation between reactive ANA and inflammatory mediators, including CRP, suggesting that the signaled inflammation may be related to the autoantibodies present. Several studies have pointed to the relationship between above-average CRP levels and autoimmune diseases such as rheumatoid arthritis and SLE (26).

#### PCT

Pro-calcitonin is a biomarker of severe systemic inflammation, particularly in bacterial infections, and elevated levels have been observed in patients with severe COVID-19. However, the role of PCT in long COVID-19 remains unclear. Although PCT is valuable for detecting bacterial infections in acute cases, its levels should generally be low in most individuals with long COVID-19, as this condition is predominantly associated with non-bacterial immune and inflammatory dysfunction. Indeed, while research indicates that elevated PCT levels is commonly associated to severe COVID-19 cases, their prognostic significance for long COVID-19 remains ambiguous (27).

In this study, 12 individuals exhibited PCT levels above 500 pg/mL. Eight of these patients were also ANA-reactive (66.67%), suggesting a correlation between autoimmune conditions and systemic inflammation, which may be a consequence of the autoimmune condition itself. This finding aligns with other analyses, indicating that elevated PCT levels can occur without bacterial pneumonia (28). In this sense, its elevation primarily reflects the inflammatory process, further implying that PCT may be involved in a broader inflammatory profile in individuals with long COVID-19.

#### Anti-CCP

Anti-cyclic citrullinated peptide antibodies have been used as a predictive marker for rheumatoid arthritis. This autoantibody can be detected 4-5 years before clinical manifestations and is present at the onset of the disease in 79% of patients, with a specificity of 98% (29).

In this study, no anti-CCP autoantibodies were found in any patient of the control or COVID group. Four patients had ANA patterns that could indicate SARDs, but the ELISA results ruled out the presence of rheumatoid arthritis. Unlike other publications that have reported significant results in the acute and even convalescent phases of COVID-19, the study by Chang et al. (7) also did not yield significant results, with anti-CCP autoantibodies identified in only 2.1% of patients.

#### RF

Rheumatoid factor comprises polyreactive antibodies that bind to the Fc region of IgG. Despite being detected in healthy individuals, RF is considered a marker for rheumatoid arthritis, with a sensitivity of 60-90%.

In the present study, two patients in the COVID-19 group were RF-positive. In one of the cases, the patient had only this altered result, while the other patient had other findings, such as reactive ANA and high CRP, suggestive of autoimmunity. Gao et al. (30) showed that the detection of RF was negative in all patients during the acute phase of severe COVID-19, demonstrating that this marker is not frequent in COVID-19 patients, even when other autoimmunity indicators are detected.

### Inferences and investigations for the development of COVID-19 autoimmunity

**Figure 3:**
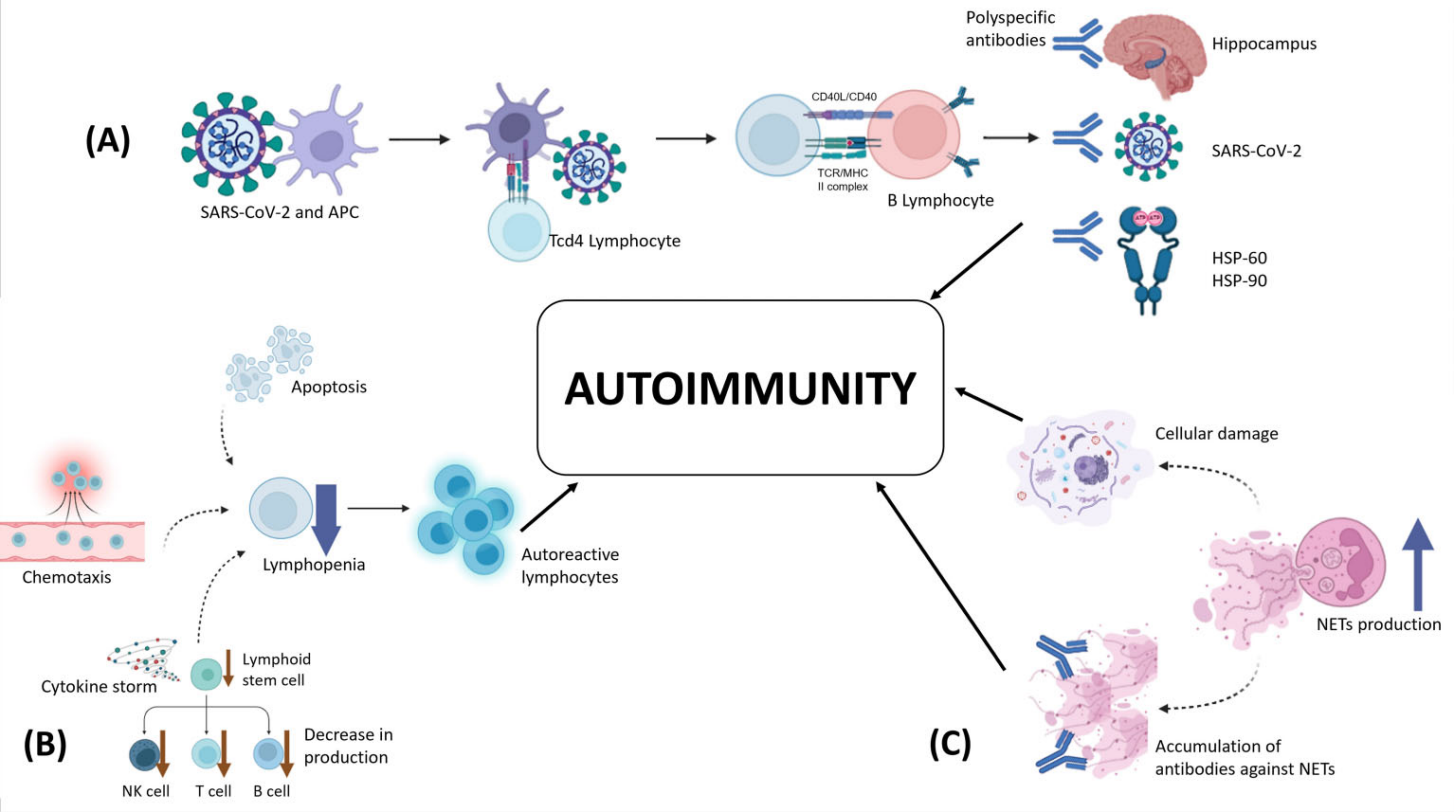
Mechanisms of autoimmunity development. **A**, Molecular mimicry. **B**, Loss of immune tolerance. **C**, Neutrophil extracellular traps (NETs). APC: antigen-presenting cell; TCR: T cell receptor; MHC II: Major histocompatibility complex class II; HSP: heat shock proteins; NET: neutrophil extracellular traps.

Figure 3 illustrates the three primary accepted mechanisms for the triggering of autoimmune responses following acute or chronic infections: molecular mimicry, temporary impairment of the immune response or loss of immune tolerance, and the action of neutrophil extracellular traps (NETs) (5,31).

#### Molecular mimicry

The molecular mimicry between virus antigens and self-antigens is a proposed explanation for the development of autoantibodies following COVID-19. Infection activates the host's immune system by presenting antigens to auxiliary lymphocytes, which stimulate B lymphocytes to produce antibodies, but these immunoglobulins are polyspecific (i.e., they can recognize multiple structurally similar antigens). Thus, the antibody that neutralizes SARS-CoV-2 proteins can recognize its own structures, causing inflammation and stimulating more autoantibody production (32).

Four main types of mimicry have been proposed: 1) self-antigen internalized by the microorganism and subsequently presented as a non-self-antigen, 2) homologous proteins between the host and invader, 3) shared amino acid sequences or epitopes, and 4) structural similarity. The types commonly associated with the development of autoimmunity are similarity and sharing of the amino acid sequence or the epitope between the virus and host (33).

An example of mimicry was reported by Liu et al. (34), in which peptides that comprise immunoreactive epitopes of SARS-CoV-2 share the same amino acid sequence as human heat shock proteins (HSPs) 60 and 90, which are associated with Guillain-Barré syndrome (GBS). This autoimmune disease affects gangliosides of the peripheral nervous system. According to Mobasheri et al. (35), the number of reported cases of GBS in Italy between March and April 2020 was five times higher than in the previous three years.

In addition, cross-reactivity between antibodies produced to neutralize virus proteins and tissues affects regions of the hippocampus, olfactory bulb, and cerebral cortex. It recognizes epitopes in smooth muscle in areas such as the lungs, kidneys, and colon. The affected regions support the main symptoms associated with long COVID, including loss of olfactory sense, memory problems, and cardiorespiratory difficulties, as patients in this condition may exhibit latent autoimmunity (36).

Also, according to Rojas et al. (33), 23 of the SARS-CoV-2 peptides were exact matches to the human proteome, six of which bind to human leukocyte antigen (HLA) proteins, suggesting the interaction between viral peptides and the major histocompatibility complex of cells as an aggravating factor in the development of autoimmunity.

The temporary impairment of the immune response occurs following transient lymphopenia after SARS-CoV-2 infection. The causes of the phenomenon are believed to include the induction of apoptosis in lymphocytes, the redistribution of immune system cells (chemotaxis), and even bone marrow failure, which is exacerbated by hyperinflammation and cytokine storm. Lymphocyte deficiency leads to a subsequent reduction in the sentinel effect - the function of lymphocytes that ensures that antibodies do not recognize their own antigens as targets, making immunological tolerance possible (5). Thus, the momentary impairment of the lymphocyte response may favor the development of autoimmunity by allowing self-reactive lymphocytes to be released to the periphery when immunity is re-established, causing a loss of tolerance to self-antigens (1).

Interestingly, Chen et al. (37) reported that mice exposed to IgG antibodies produced by patients with long COVID-19 developed behavioral symptoms. The authors also showed an increase in IgG in the tissues analyzed, such as muscle, indicating the role of autoantibodies in the development of long COVID-19, which corroborates the hypothesis of loss of immune tolerance.

NETs are networks composed of chromatin, microbicidal proteins, and oxidizing enzymes, which are released to contain infections once neutrophils are activated either by inflammatory cytokines or a virus entering the cell (38). However, according to Veras et al. (39), neutrophils infected with SARS-CoV-2 released more NETs than uninfected neutrophils.

It has also been shown that patients with long COVID-19 still have higher levels of NET after clinical recovery, indicating incomplete resolution of the condition after the acute phase of the infection. One explanation might be the presence of IgG and IgM autoantibodies against NETs in patients infected with SARS-CoV-2, which protects them from enzymatic degradation, as the ability of DNAse to degrade NETs is inversely proportional to the presence of these autoantibodies. Interestingly, patients with long COVID-19 often express detectable levels of ANA even 12 months after the acute infection, which are related to anti-NET antibodies (31).

Additionally, neutrophils contribute to autoimmunity by causing collateral tissue damage. In this case, excess enzymes and toxins in the NETs can damage healthy tissues, releasing unusual self-antigens that were not presented, which are recognized as foreign by the immune system, triggering an autoimmune response (40).

### Limitations

This study has some limitations that should be acknowledged. Notably, the ANA and VDRL tests used are screening tools and, on their own, are insufficient for the definitive diagnosis of autoimmune diseases. Confirmatory follow-up tests would be necessary in individuals with ANA reactivity to establish specific autoimmune conditions. However, the wide variety of ANA fluorescence patterns observed in this study suggested multiple potential autoimmune disease pathways, including systemic lupus erythematosus, systemic sclerosis, and autoimmune myositis. This heterogeneity limited the feasibility of performing targeted confirmatory tests for each suspected condition within the scope of the study. Future research should include extended serological panels and clinical follow-up to better characterize the autoimmune profiles of these patients.

## Conclusions

In summary, the ANA and VDRL results reported in this study suggest the presence of autoantibodies in patients who have had severe COVID-19 requiring hospitalization. Despite being sensitive screening tests, increased levels of these autoantibodies were not detected in patients who had no contact with the virus. Moreover, the altered CRP and PCT results indicate a persistent inflammatory state, which can favor the development of autoantibodies in the long COVID condition. Thus, these patients may be more susceptible to the manifestations of autoimmune diseases in the future than individuals not infected with the SARS-CoV-2 virus.

According to the results of this study, which are in agreement with other acute and long-term COVID-19 research, the symptoms of COVID-19 may be related to the development of autoimmunity after infection, a phenomenon characterized by elevated inflammatory markers and autoantibodies.

## Data Availability

All data generated or analyzed during this study are included in this published article

## References

[1] Yong SJ (2021). Long COVID or post-COVID-19 syndrome: putative pathophysiology, risk factors, and treatments. Infect Dis (Lond).

[2] Davis HE, McCorkell L, Vogel JM, Topol EJ (2023). Long COVID: major findings, mechanisms and recommendations. Nat Rev Microbiol.

[3] Ehrenfeld M, Tincani A, Andreoli L, Cattalini M, Greenbaum A, Kanduc D (2020). Covid-19 and autoimmunity. Autoimmun Rev.

[4] Stoian M, Procopiescu B, Șeitan S, Scarlat G (2023). Post-COVID-19 syndrome: Insights into a novel post-infectious systemic disorder. J Med Life.

[5] Caãas CA (2020). The triggering of post-COVID-19 autoimmunity phenomena could be associated with both transient immunosuppression and an inappropriate form of immune reconstitution in susceptible individuals. Medi Hypotheses.

[6] Liu F, Li L, Xu M, Wu J, Luo D, Zhu Y (2020). Prognostic value of interleukin-6, C-reactive protein, and procalcitonin in patients with COVID-19. J Clin Virol.

[7] Chang SH, Minn D, Kim YK (2021). Autoantibodies in moderate and critical cases of COVID-19. Clin Transl Sci.

[8] Tesch F, Ehm F, Vivirito A, Wende D, Batram M, Loser F (2023). Incident autoimmune diseases in association with SARS-CoV-2 infection: a matched cohort study. Clin Rheumatol.

[9] Dellavance A, Leser PG, Andrade LEC (2007). Análise crítica do teste de anticorpos antinúcleo (FAn) na prática clínica. Rev Bras Reumatol.

[10] Pascolini S, Vannini A, Deleonardi G, Ciordinik M, Sensoli A, Carletti I (2021). COVID-19 and immunological dysregulation: can autoantibodies be useful?. Clin Transl Sci.

[11] İnal N, Kurumanastırlı B, Taşkınoğlu T, Duran A, Togay A, Sarıgüzel FM (2024). Retrospective evaluation of “Rods and Rings” pattern detected in the anti-nuclear antibody (ANA) indirect immunofluorescence (IIF) test. Front Immunol.

[12] Chandra A, Kahaleh B (2022). Systemic sclerosis (SSc) after COVID-19: a case report. Cureus.

[13] Wahab AA, Jauhary EJ, Ding CH (2023). The recognition of anti-nuclear antibody's dense fine speckled pattern and the detection of anti-DFS70 antibodies in the laboratory practice: Its prevalence and clinical significance. Malays J Pathol.

[14] Arcani R, Bertin D, Bardin N, Mazodier K, Jean R, Suchon P (2021). Anti-NuMA antibodies: clinical associations and significance in patients with primary Sjögren's syndrome or systemic lupus erythematosus. Rheumatology (Oxford).

[15] Phillips B, Martin J, Rhys-Dillon C (2022). Correction to: Increased incidence of anti-synthetase syndrome during COVID-19 pandemic. Rheumatology (Oxford).

[16] von Mühlen CA, Garcia-De La Torre I, Infantino M, Damoiseaux J, Andrade LEC, Carballo OG (2021). How to report the antinuclear antibodies (anti-cell antibodies) test on HEp-2 cells: guidelines from the ICAP initiative. Immunol Res.

[17] He J, Wei X, Sturgess A (2022). Concordance between myositis autoantibodies and anti-nuclear antibody patterns in a real-world, Australian cohort. Rheumatology (Oxford).

[18] Araújo FC, Amaral ACD, Silva HJ, Santos JNV, Mendonça VA, Oliveira VC (2025). Autoantibodies as potential prognostic factors for clinical outcomes related to COVID-19: a systematic review of inception prospective cohort studies with GRADE recommendations. Braz J Med Biol Res.

[19] Al Attia HM (2002). False positive VDRL (BFP-STS) and systemic lupus erythematosus; new data in clinico-laboratory associations. Int J Dermatol.

[20] Butt A, Erkan D, Lee AI (2022). COVID-19 and antiphospholipid antibodies. Best Pract Res Clin Haematol.

[21] Altmann DM, Whettlock EM, Liu S, Arachchillage DJ, Boyton RJ (2023). The immunology of long COVID. Nat Rev Immunol.

[22] Ahmed G, George CA, Ganguly S (2022). Post-COVID-19 biologically false-positive VDRL: A report. Int J STD AIDS.

[23] Hara S, Sanatani T, Tachikawa N, Yoshimura Y, Miyata N, Sasaki N (2022). Comparison of the levels of neopterin, CRP, and IL-6 in patients infected with and without SARS-CoV-2. Heliyon.

[24] Levinson T, Wasserman A, Shenhar-Tsarfaty S, Halutz O, Shapira I, Zeltser D (2023). Comparative analysis of CRP as a biomarker of the inflammatory response intensity among common viral infections affecting the lungs: COVID-19 versus influenza A, influenza B and respiratory syncytial virus. Clin Exp Med.

[25] Son K, Jamil R, Chowdhury A, Mukherjee M, Venegas C, Miyasaki K (2023). Circulating anti-nuclear autoantibodies in COVID-19 survivors predict long COVID symptoms. Eur Respir J.

[26] Li Y, Zhang S, Liu J, Zhang Y, Zhang N, Cheng Q (2023). The pentraxin family in autoimmune disease. Clin Chim Acta.

[27] Muflihah H, Rahiman SB, Sastramihardja HS, Yulianto FA (2022). Procalcitonin, but not D-Dimer, is an inapplicable biomarker for severe COVID-19. Kne Life Sci.

[28] Roy A, Powers HR, Craver EC, Nazareno MD, Yarrarapu SNS, Sanghavi D (2021). Antibiotic Stewardship: early discontinuation of antibiotics based on procalcitonin level in COVID‐19 pneumonia. J Clin Pharm Ther.

[29] Roghani SA, Dastbaz M, Lotfi R, Shamsi A, Abdan Z, Rostampour R (2024). The development of anticyclic citrullinated peptide (anti-CCP) antibody following severe COVID-19. Immun Inflamm Dis.

[30] Gao ZW, Wang X, Lin F, Dong K (2020). The correlation between SARS-CoV-2 infection and rheumatic disease. Autoimmun Rev.

[31] Shafqat A, Omer MH, Albalkhi I, Alabdul Razzak G, Abdulkader H, Abdul R (2023). Neutrophil extracellular traps and long COVID. Front Immunol.

[32] Proal AD, VanElzakker MB (2021). Long COVID or Post-acute sequelae of COVID-19 (PASC): an overview of biological factors that may contribute to persistent symptoms. Front Microbiol.

[33] Rojas M, Herrán M, Ramírez-Santana C, Leung PSC, Anaya JM, Ridgway WM (2023). Molecular mimicry and autoimmunity in the time of COVID-19. J Autoimmun.

[34] Liu Y, Sawalha AH, Lu Q (2021). COVID-19 and autoimmune diseases. Curr Opin Rheumatol.

[35] Mobasheri L, Nasirpour MH, Masoumi E, Azarnaminy AF, Jafari M, Esmaeili SA (2022). SARS-CoV-2 triggering autoimmune diseases. Cytokine.

[36] Kreye J, Reincke SM, Kornau HC, Sánchez-Sendin E, Corman VM, Liu H (2020). A therapeutic non-self-reactive SARS-CoV-2 antibody protects from lung pathology in a COVID-19 hamster model. Cell.

[37] Chen HJ, Appelman B, Willemen H, Bos A, Prado J, Geyer CE (2024). Transfer of IgG from long COVID patients induces symptomology in mice. bioRxiv.

[38] Zuo Y, Yalavarthi S, Shi H, Gockman K, Zuo M, Madison JA (2020). Neutrophil extracellular traps in COVID-19. JCI Insight.

[39] Veras FP, Pontelli MC, Silva CM, Toller-Kawahisa JE, de Lima M, Nascimento DC (2020). SARS-CoV-2-triggered neutrophil extracellular traps mediate COVID-19 pathology. J Exp Med.

[40] Fallahi P, Elia G, Ragusa F, Paparo SR, Patrizio A, Balestri E (2023). Thyroid autoimmunity and SARS-CoV-2 infection. J Clin Med.

